# New Pathological Lesions Developed in Pigs by a “Non-virulent” Strain of *Glaesserella parasuis*

**DOI:** 10.3389/fvets.2020.00098

**Published:** 2020-02-25

**Authors:** Cláudia Cerutti Dazzi, João Antônio Guizzo, Simone Ramos Prigol, Luiz Carlos Kreutz, David Driemeier, Somshukla Chaudhuri, Anthony Bernard Schryvers, Rafael Frandoloso

**Affiliations:** ^1^Laboratory of Microbiology and Advanced Immunology, Faculty of Agronomy and Veterinary Medicine, University of Passo Fundo, Passo Fundo, Brazil; ^2^Department of Veterinary Pathology, School of Veterinary Medicine, Federal University of Rio Grande do Sul, Porto Alegre, Brazil; ^3^Department of Microbiology and Infectious Diseases, Faculty of Medicine, University of Calgary, Calgary, AB, Canada

**Keywords:** *Glaesserella parasuis*, serovar 7, Glässer's disease, clinical signs, pathology

## Abstract

*Glaesserella parasuis* is a Gram-negative bacterium that causes Glässer's disease, a common pathology found in young pigs characterized by polyarthritis, polyserositis, and meningitis. The bacterium has 15 known serovars that have been classified by virulence. Serovars 1, 4, 5, and 12 are considered highly virulent and used in most studies. Serovars 3, 6, 7, 9, and 11 are considered avirulent. Recent reports that serovar 7 is an emerging problem in the pig industry indicate that the association of virulence and serovar may not always be reliable. This led us to infect colostrum-deprived piglets with the reference serovar 7 strain (SV7 strain 174) that had been passaged through pigs and characterize the clinical and pathological signs. We observed that SV7 strain 174 caused clinical signs consistent with Glässer's disease in all infected piglets that succumbed to infection for up to day 5 post-infection. Macroscopic and microscopic lesions were consistent with those found in piglets infected with conventional virulent serovars. In addition, we describe novel microscopic lesions associated with Glässer's disease such as endophthalmitis and thymic depletion. Thus, our findings indicate that SV7 strain 174 causes classical signs of Glässer's disease in colostrum-deprived piglets and some caution should be used in employing vaccine strategies based on association between capsular serovar and virulence.

## Introduction

Glässer's disease (GD) is an emergent and worldwide disease in pigs caused by *Glaesserella parasuis* (*G. parasuis*), formerly known as *Haemophilus parasuis*, a Gram-negative bacterium that belongs to the *Pasteurelaceae* family. Clinically, GD is characterized by polyserositis, polyarthritis and meningitis (1) and mainly affects young pigs in the nursery phase. A serotyping scheme was originally developed to identify lineages of bacteria by their extracellular polysaccharide capsule and were classified as non-virulent, moderately virulent and highly virulent according to their ability to cause disease after intraperitoneal inoculation in specific pathogen-free pigs (2). Strains representing serovars 1, 5, 10, 12, 13, and 14 were deemed highly virulent, whereas strains representing serovars 2, 4, 8, and 15 were characterized as moderately virulent and strains representing serovars 3, 6, 7, 9, and 11 were considered avirulent.

In the original study evaluating virulence the standard *G. parasuis* serovar 7 strain (174) was unable to cause disease in piglets after an intraperitoneal challenge (2). A subsequent study involving intranasal challenge of highly susceptible piglets confirmed the inability of this strain to cause infection (3). However, there have been recent outbreaks of GD with SV7 in swine herds in China (4) and Brazil (5). In Australia, nasal samples obtained from pigs showing clinical signs typical of GD were positive for SV7 *G. parasuis* (6). These observations raise questions as to whether it is still reasonable to characterize all strains that express the serovar 7 capsule as avirulent.

Reproducing GD can be challenging and has primarily been performed in an animal model with highly susceptible pigs (specific pathogen-free, cesarean-derived, colostrum deprived pigs and natural farrow, colostrum-deprived pigs). The experiments have primarily been performed with strains previously classified as virulent (SV1, 5, and 12) or moderately virulent (SV4) (7–9). Intratracheal inoculation in these piglets can lead to a systemic infection that is much more rapidly progressive than natural GD thus full protection that is achieved by vaccination in this model (10) is likely to translate into full protection in the commercial sphere. However, the protective effect of immunization on earlier stages of naturally occurring disease is not evaluated with this model.

The clinical signs commonly observed in pigs infected with virulent *G. parasuis* strains include rough hair, fever, joint swelling, and lameness as well as signs associated with respiratory difficulties such as coughing, sneezing, dyspnea or tachypnea, the latter of which can progress to neurological signs and death (8). Common pathological findings upon necropsy are fibrinoid arthritis, peritonitis, pleuritis, pericarditis, and meningitis (7). Microscopically, these lesions are characterized by the presence of fibrinosuppurative material on affected organs. Sometimes meningitis is seen only by microscopic analysis. Besides fibrinosuppurative polyserositis, interstitial pneumonia, or fibrinosuppurative bronchopneumonia can also be seen in the lungs. Disseminated intravascular coagulation, vascular thrombosis, hemorrhages, congestion, and edema in different organs (mostly lungs and brain) may be present (9). In acute cases, septic shock manifestations are the most common observation (7).

In this study, we considered the possibility that stocks of strains may contain variants with reduced virulence and that passage of strains in the laboratory and between laboratories could result in further reduction in virulence. In addition, starting cultures for challenge experiments with individual colonies could potentially raise the risk of producing a non-virulent challenge preparation. Thus, we passaged *G. parasuis* serovar 7 strain 174 in pigs and prepared frozen stocks that would be used to prepare cultures for challenge experiments. Using this approach, we performed a challenge experiment in naturally farrowed colostrum-deprived pigs and present clinical and pathological lesions including microscopic lesions that have not been previously described in a case of *G. parasuis* infection.

## Materials and Methods

### Preparation of a Standard Challenge Inoculum

We obtained a frozen stock of *G. parasuis* strain 174, the representative SV7 strain, from Dr. Rodriguez-Ferri, that was reported to have only been passaged 5 times from the original stock (2). A sample from the culture was streaked onto chocolate plates and a sample of the solid section of the resulting growth was used to inoculate supplemented PPLO broth [60 μg/mL nicotinamide adenine dinucleotide (β-NAD, Sigma-Adrich, USA) and 2.5 mg/mL D-glucose (Sigma-Aldrich, USA)] and incubated with shaking (250 rpm/37°C) until it reached optical density of 0.4 at 600 nm. The bacteria were collected by centrifugation, washed twice in PBS [137 mM NaCl (Sigma-Aldrich, USA), 2.7 mM KCl (Sigma-Aldrich, USA), 10 mM Na_2_HPO_4_ (Sigma-Aldrich, USA), 1.8 mM KH_2_PO_4_ (Sigma-Aldrich) pH 7.2] and counted using Flow Cytometry (FACSVerse Cytometer–Becton Dickinson, USA) to quantify the challenge inoculum. Inocula were prepared at 3 × 10^8^ bacteria in a final volume of 2 mL of RPMI 1640 medium (Invitrogen, USA). The bacteria were introduced into the lungs of 6 colostrum deprived piglets via intratracheal injection. The piglets were negative for *G. parasuis, Actinobacillus pleuropneumoniae, Pasteurella multocida, Mycoplasma hyopneumoniae*, and Porcine Circovirus type 2 by molecular and serological tests.

Piglets were monitored by fever, joint swelling, lameness, dyspnea, tachypnea, coughing, sneezing and neurological signs and euthanized by intracardiac injection of 0.3 mL/kg of embutramide (MSD, Intervet GmbH, Germany) under anesthesia (protocol described below) when the animals showed severe clinical signs. The sample from the brain tissue of an infected pig was plated on chocolate agar plates and all the colonies presumptively identified as *G. parasuis*, with genomic DNA from a single colony confirmed by Real Time PCR (6). The colonies from the brain tissue were pooled and used to inoculate supplemented PPLO broth and grown to an optical density of 0.7, and the cell density determined by flow cytometry and distributed in 1 mL aliquots of frozen medium [50 g/L of Skim Milk (Sigma-Aldrich, USA), 25 g/L of Tryptone (Sigma-Aldrich, USA) and 20% of glycerol (Sigma-Aldrich, USA)] and stored frozen at −80°C. The purity of the pooled culture was confirmed by Gram staining and negative PCR analysis for *A. pleuropneumoniae, P. multocida, B. bronchispetica, M. hyopneumoniae, M. hyorhinis, M. hyosynoviae* but positive for *G. parasuis*. The capsular type of the pooled stock was determined by multiplex PCR using the strategy described by Espindola et al. (11).

The frozen stock was used to inoculate 100 mLs of pre-warmed, supplemented PPLO broth and incubated with shaking until mid-log phase (0.6 A 600 nm), centrifuged (4.500 rpm for 20 min) and resuspended in 10 mL RPMI 1640 (Invitrogen, USA). The cell count was quantified by flow cytometer as described above, and then diluted to 1 × 10^7^ cells per 2 mL of RPMI 1640 which was directly injected into the trachea.

### Pig Experimental Infection and Clinical Evaluation

Twenty snatch-farrowed colostrum-deprived piglets (DB Genética Suína, Brazil) selected from five different sows from a high health status herd were obtained and raised as described previously (12). At day 42, piglets were anesthetized using the following drug combination: 0.3 mg/kg of acepromazine (Syntec do Brasil, Brazil), 0.3 mg/kg of midazolam (Laboratório Teuto Brasileiro, Brazil), and 15 mg/kg of Ketamine (Ceva Santé Animale, Brazil) injected by the intramuscular route. *G. parasuis* challenge was performed via the intratracheal route with 1 × 10^7^ *H. parasuis* serovar 7 strain 174 prepared as described in the previous section. Prior to challenge, rectal temperature and the perimeter of the radio-humeral, carpal, hock and tarsal—right and left joints were measured (cm) in all animals with a measuring tape. Following the challenge, rectal temperatures and clinical signs such as joint swelling, lameness, dyspnea, tachypnea, coughing, sneezing, and neurological signs were assessed at 12-h intervals until the animal's death. When signs were too severe or the animals demonstrated signs of suffering, they were anesthetized with the same drug combination used for the challenge and then euthanized by intracardiac injection of 0.3 mL/kg of embutramide (MSD, Intervet GmbH, Germany).

### Necropsy and Sampling

Necropsy was immediately performed and documented following the death of an animal. The post-mortem perimeter of the joints was measured. The carcass was laid in dorsal decubitus and the members were rebated. Sterile incisions in the abdominal and thoracic cavities were made for bacteriological sampling, followed by the pericardium, central nervous system, and articulations. After sampling, the ribs and sternum were removed to expose the lungs and heart and abdominal cavity. The organs were removed, first the abdominal organs followed by the thoracic organs. The head skin was removed exposing frontal, parietal and occipital bones; a hacksaw was flame-sterilized and used to crack the bones in a triangle incision trough frontal and lateral borders. After removing the skullcap, an incision was made through the meninges with a sterile scalp blade to collect swabs for bacteriological assay. Macroscopic and microscopic lesions were scored as no alterations, mild, moderate, or accentuated changes.

### Microbial Analysis

Swab samples taken during necropsy were seeded onto chocolate agar plates and incubated in a microaerophilic environment (37°C under 5% CO2) for 24–48 h. *G. parasuis* colonies were isolated from chocolate agar plates and suspended in 100 μL of ultrapure water (Sigma-Aldrich, USA). The samples were heated at 95°C for 10 min and centrifuged at 13.000 rpm for 10 min. The supernatant containing the DNA was collected and analyzed by spectrophotometer (NanoDrop, Thermo Scientific, USA) prior to performing multiplex PCR (13) to determine the serovar.

### Histology

Fragments of all organs were collected, fixed in 10% buffered formalin, processed in an automatic tissue processer (PT09, Lupetec, Brazil), paraffin embedded, sectioned at 5 μm and stained with hematoxylin and eosin (H&E) for histological analysis. Microscopic lesions were scored as mild changes (+), moderate changes (++) and accentuated changes (+++).

### Immunohistochemistry

Primary antiserum was developed in New Zealand White Rabbit using a hyper-immunization protocol. Two animals were immunized five times (days 0, 14, 28, 35, and 42) by subcutaneous injection using an inactivated (formalized) vaccine formulated with 1 × 10^9^ *G. parasuis* serovar 7 strain 174 and adjuvanted with 20% (v/v) of Montanide Gel 01 (Seppic, France). Fifteen days after the last immunization, the animals were bled and the sera was adsorbed against *A. pleuropneumoniae, Streptococcus suis* and *P. multocida* (5 × 10^9^ bacteria per 1 mL of sera) and stored at −80°C till needed.

The 3 μm tissue sections (thymus and eyes) from pigs were placed on coated glass slides (StarFrost®-Microscope Slides Advanced Adhesive), deparaffinized in three xylene baths for 10 min, then hydrated in baths with decreasing levels of alcohol (100–70%) and then transferred to distilled water. Endogenous peroxidases were inhibited in 3% hydrogen peroxide (in methanol) bath for 20 min, followed by antigen retrieval by proteinase grade XIV 0,05% (Sigma-Aldrich, USA) for 20 min at 37°C. Non-specific reactions were blocked by 5% skim milk bath for 40 min. The primary antibody was diluted 1:200 in PBS and incubated overnight in a moist chamber at room temperature. The detection system used was MACH 4 Universal HRP-Polymer (Biocare Medical, USA) according to the manufacturer's instructions. The sections were counterstained with Mayer's hematoxylin and the chromogen was applied for 1.5 min (3-amino-9-ethylcarbazol, AEC, DakoCytomation). Finally, the slides were covered with coverslips under aqueous medium.

### Mouse Experimental Infection

Twenty C57BL/6 mice (Charles River Laboratories, Saint-Constant, QC, Canada) of 8 weeks of age were randomly assigned to five groups of four mice and housed in separated cages. Animals were maintained on a 12:12-h light:dark cycle with water and sterilized feed *ad libitum*. After 1 week of acclimatization, four groups of mice were challenged intraperitoneally (IP) with four different concentration (1 × 10^8^, 1 × 10^9^, 3 × 10^9^, and 5 × 10^9^) of *G. parasuis* strain 174. Previous to the challenge, all mice received one single dose of 3.2 mg of iron dextran (Sigma-Aldrich, USA) by intraperitoneal injection. The fifth group received only the iron dextran injection. After the challenge, the animals were clinically evaluated every 4 h during the first day of the experiment and after, 3 times per day until the end of 1 week. Animals demonstrated signs of suffering or sepsis, such as depression, rough hair, prostration, lethargy, and swollen eyes were immediately euthanized using CO_2_ chamber followed by cervical dislocation.

### Ethics Statement

The pig experiment was approved by the Institutional Committee for Ethical Use of Animals of the University of Passo Fundo (protocol no. 018/2016) and followed the Brazilian College of Animal Experimentation guidelines. The mouse experiment was conducted in accordance with the guidelines of the Canadian Council on Animal Care. The study protocol was approved by the Animal Care Committee of the University of Calgary (AC15-0006).

## Results

### Preparation of a Standard Challenge Inoculum

Recognizing that serial passage of clinical isolates could ultimately result in loss of virulence we started with a stock that had reportedly only been passaged five times from stocks obtained from the original study (2). Since this stock could potentially be heterogenous with respect to the virulence of the bacteria present, we streaked the stock onto chocolate plates and used a section of the resulting culture that would likely represent the entire bacterial population to prepare a stock to be used for an intratracheal challenge in colostrum-deprived piglets. The rationale for this experiment is that during the challenge either pre-existing virulent isolates would outgrow the less virulent strains or virulent isolates generated during *in vivo* growth would selectively be responsible for infecting various tissues during systemic spread.

We infected 6 colostrum-deprived, 28-day old piglets (7.3 ± 1.1 Kg average weight) with 3 × 10^8^ *G. parasuis* by intratracheal injection and monitored the piglets for clinical signs. The piglets had a relatively rapid course of infection with clinical signs suggesting that systemic infection was occurring. The piglets were euthanized and upon necropsy, and samples from various tissues were plated onto chocolate agar plates. The sample from brain tissue of a piglet that had the most rapid and serious course of infection was selected for generating a stock of *G. parasuis* strain 174 (SV7) as described in the methods section. One mL aliquots of the stock containing 3 × 10^8^ bacteria resuspended in frozen medium were stored at −80°C and used as a starting inoculum in subsequent experiments.

### Testing the Standard Inoculum in Pigs

Although PCR analysis (13) indicated that our stock contained *G. parasuis* serovar 7 strain it was important to determine whether the disease induced by the isolate we obtained after passage in an infected pig was able to reproducibly cause the typical GD disease. Thus, the standard stock was used to prepare inoculum for intratracheal administration to 20 pigs from 5 different sows. An inoculum containing 1 × 10^7^ bacteria was administered by intratracheal injection and the piglets were monitored every 12 h for clinical signs.

**Figure 1:**
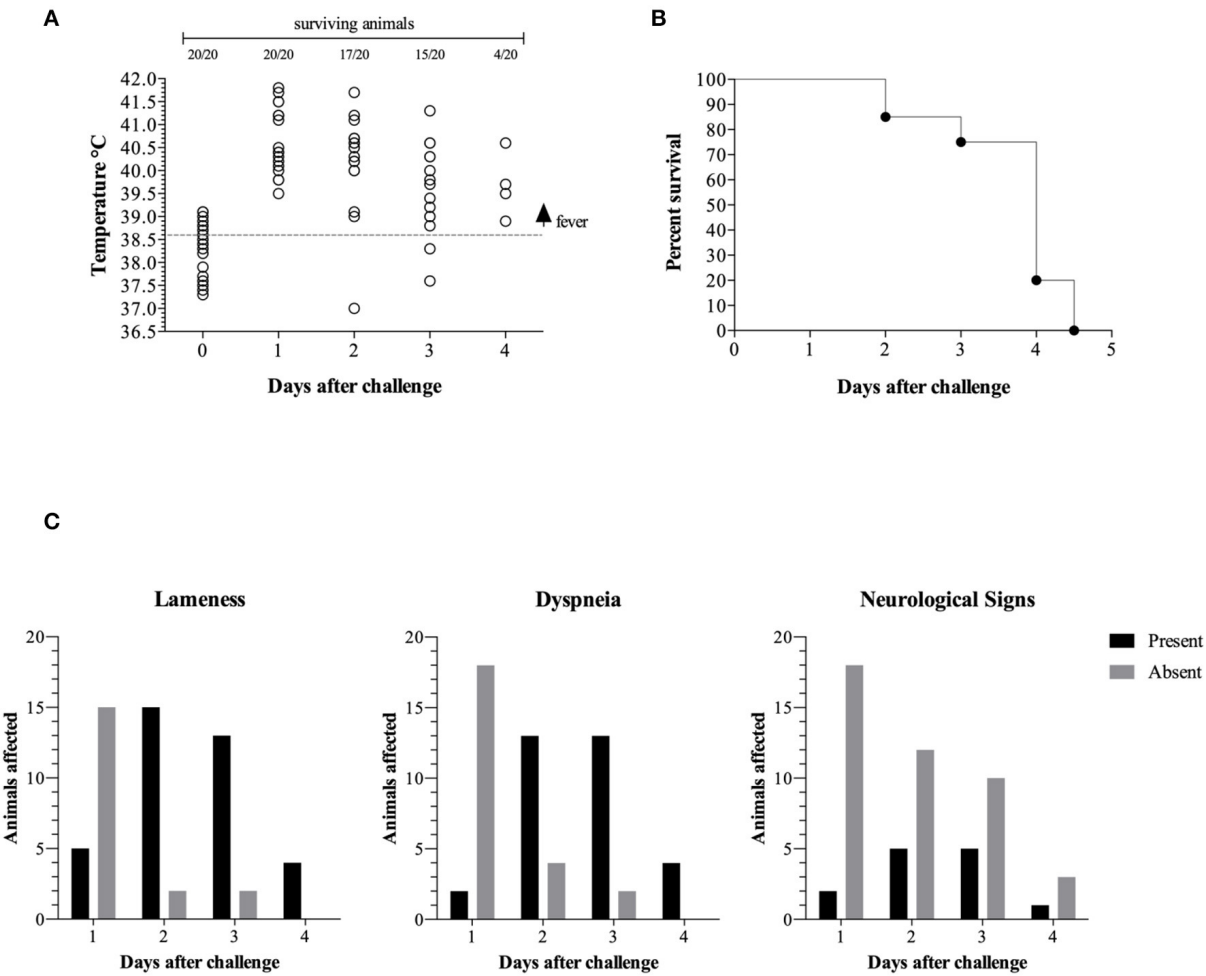
Clinical progression is represented by temperature vs. days post challenge (dpc). The gray dotted line represents the fever threshold (39.6°C) **(A)**. Pigs surviving challenge **(B)**. Clinical signs in surviving animals **(C)**.

At day 1 post challenge all animals presented with a fever above 39.5°C and most piglets remained febrile until they reached endpoint (Figure 1A). We also observed signs of apathy and decreased appetite (hyporexia) from the first day post challenge in all the pigs. On the second and the third day post-challenge all surviving piglets were apathetic with anorexia or hyporexia and cyanosis with some pigs having cold extremities.

The majority of the surviving animals displayed lameness on day 2 (15/17), day 3 (13/15), and day 4 (4/4) post challenge (Figures 1B,C). Similarly, the majority of the surviving piglets displayed difficulty breathing (dyspnea) on day 2 (13/17), day 3 (13/15), and day 4 (4/4) post challenge. A subset of the surviving piglets displayed neurological signs on day 2 (5/17), day 3 (5/15), and day 4 (1/4) post challenge.

These results indicate that the piglets challenged by intratracheal injection of 1 × 10^7^ *G. parasuis* strain 174 (SV7) prepared from our standard stock displayed the expected signs of Glässer's disease.

### Macroscopic Lesions

The classical lesions of Glässer's disease (GD) present in the piglets at necropsy are listed in Table 1. Nineteen animals presented with typical GD lesions, with the sole exception being a piglet that died within the first 24 h after challenge. This piglet had petechiae in the epicardium, lungs, and thymus. The tissue samples from all piglets were positive for growth of *G. parasuis*, except the peritoneal swab from piglet n° 8 which at necropsy had no signs of fibrin deposition or liquid alterations.

**Table 1:** Main pathological findings and intensity according to the day of death post challenge.

**Findings**	**Pig number**																			
	**01**	**02**	**03**	**04**	**05**	**06**	**07**	**08**	**09**	**10**	**11**	**12**	**13**	**14**	**15**	**16**	**17**	**18**	**19**	**20**
Death (dpc)	4^th^	3^rd^	4^th^	1^st^	4^th^	4^th^	4^th^	2^nd^	3^rd^	4^th^	5^th^	4^th^	4^th^	2^nd^	5^th^	5^th^	4^th^	4^th^	4^th^	4^th^
Pleuritis	+++	+	Ø	Ø	Ø	++	+	+	+	+	++	++	+	+	+	+	+++	Ø	++	+++
Pericarditis	+	+++	++	Ø	++	+	++	Ø	+++	+++	++	++	++	+++	++	+	+++	+++	++	+++
Polyarthritis	++	+	++	Ø	+	+	+++	Ø	++	+++	++	+++	Ø	++	+++	+++	++	+++	+++	++
Peritonitis	Ø	+	+++	Ø	Ø	+	++	Ø	++	++	++	Ø	++	+	+	Ø	+++	+++	+++	++
Meningitis	+++	++	Ø	Ø	+++	+	++	Ø	+	+++	Ø	+++	+++	Ø	Ø	+++	+	++	Ø	+

*Ø, none; +, mild; ++, moderate; +++, accentuated*.

**Figure 2:**
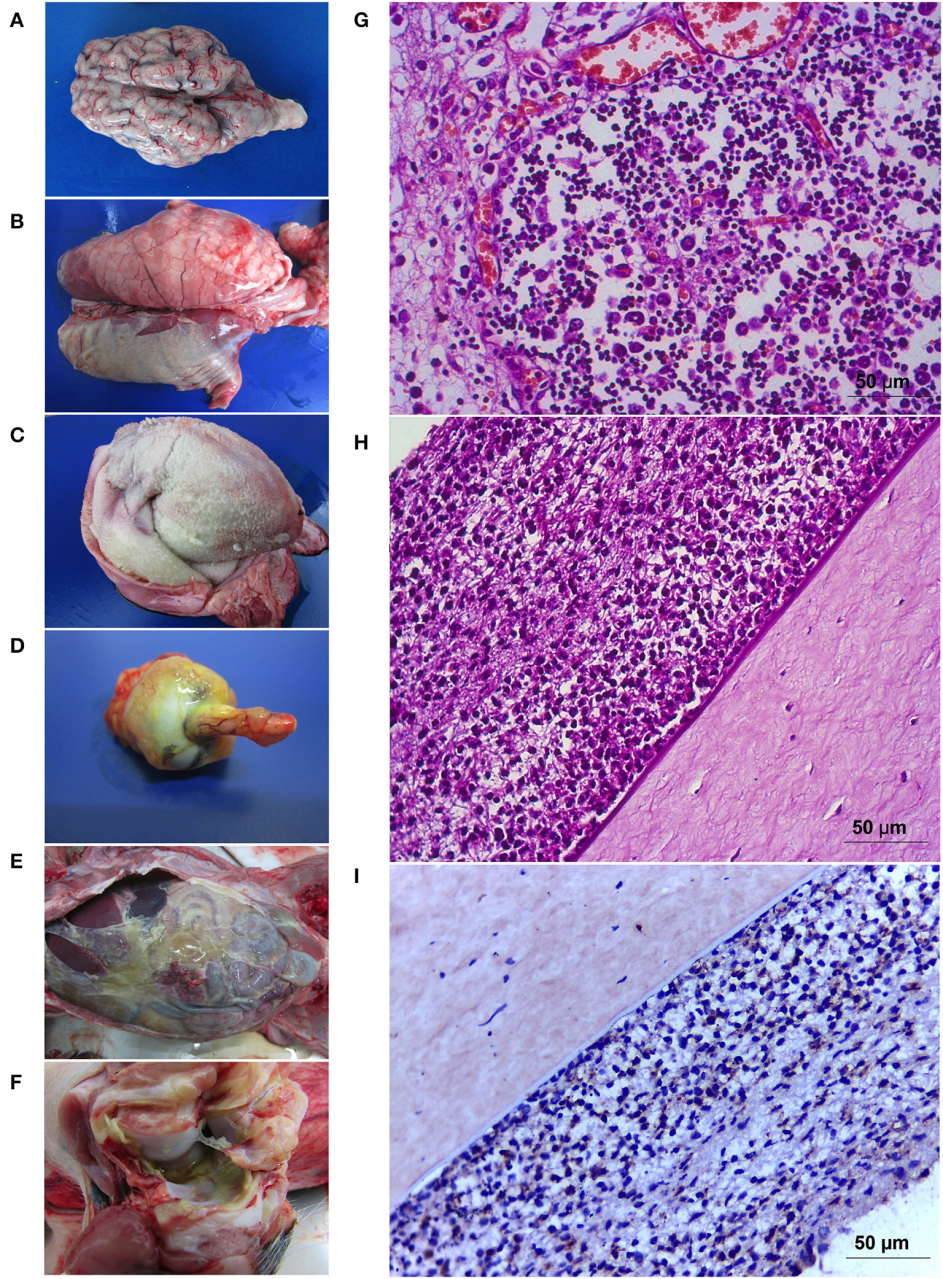
Pathology caused by *Glaesserella parasuis* SV7 strain 174. Gross pathology illustrating meningitis **(A)** that is prominent in the cerebellum in some cases. Severe pleuritis **(B)**, fibrinosuppurative pericarditis **(C)**, corneal opacity associated with congestion of the optic nerve **(D)** in endophthalmitis cases. Peritonitis with a dense yellowish fibrin content covered the abdominal cavity **(E)** and arthritis **(F)**. Tissue sections showing the cortical zone of thymus with a marked lymphoid depletion **(G)**. Iris and ciliary body contained moderate mixed infiltrate (mostly neutrophils and macrophages) **(H)** with positive detection for *G. parasuis* in the immunostaining **(I)**.

At necropsy 16 piglets had varying levels of pleuritis (Table 1) whereas 18 piglets had varying levels of pericarditis (Figure 2C). Pleuritis was observed in 16 piglets (Figure 2B) and polyarthritis was present in 17 piglets (Figure 2F). Peritonitis was only observed in 14 piglets (Figure 2E). Meningitis was only present in 13 piglets with varying levels of involvement (Figure 2A). Four animals (n° 7, 10, 13, and 18) presented corneal opacity with scleral congestion and optical nerve congestion (Figure 2D) which has not been reported previously.

### Microscopic Lesions and Immunohistochemical Characterization

Polyserositis was characterized by the deposition of delicate fibrin nets associated with the presence of abundant neutrophils and macrophages with cytoplasm filled with granular basophilic material, resembling coccobacilli bacteria. Lungs also presented with suppurative multifocal interstitial or bronchointerstitial pneumonia with interlobular septa distention with fibrinosuppurative content. Mediastinal and mesenteric lymph nodes had suppurative lymphadenitis, with some having associated lymphoid depletion. The spleen of most animals presented with white pulp depletion, with or without lympholysis and, discrete red pulp depletion. Kidneys, liver, and pancreas had discrete cell infiltrate composed mostly by mononuclear cells and sparse neutrophils, characterizing discrete pyelitis, nephritis, pancreatitis and, hepatitis. Fibrinosuppurative meningitis was the main lesion in the central nervous system and was seen in 13 animals with different intensities varying from discrete to severe. Pig number 1 and 6 had accentuated focal suppurative leukoencephalomalacia near lateral ventricle. Four animals (number 7, 10, 13, and 18) with meningitis had optical nerve fibrinosuppurative perineuritis and corneal degeneration. Animals number 10 and 13 also had abundant neutrophils and fibrinoid material admixed within conjunctiva and attached to corneal epithelium. The iris and ciliary body contained moderate mixed infiltrate (mostly neutrophils and macrophages); these cells exfoliated into the anterior and posterior chambers, associated with fibrin, and adhered to corneal endothelium (Figure 2H). The same content was seen in filtration angle and trabecular meshwork. Neutrophils were seen in choroid, posterior cavity and vitreous humor, and associated in the surface retinal layer. Congestion of retinal vessels were a prominent finding. The inflammation of all those ocular segments characterizes endophthalmitis. The thymus in all animals presented moderate to accentuated lymphoid depletion in a starry sky pattern, some of them presenting loss of the corticomedullar ratio and atrophy and were associated with accentuated fibrinosuppurative serositis (Figure 2G). Immunohistochemistry revealed ocular sections lesion sites with cellular infiltrate (Figure 2I), thymic fibrinoid lesions showed positive in the immunostaining for *G. parasuis*.

### Testing the Standard Inoculum in Mice

Mice are used extensively for evaluating potential vaccine candidates against human pathogens and can serve as a less expensive initial screening system for testing vaccine candidates against animal pathogens. Previous reports of using mice for evaluating vaccine candidates against *G. parasuis* in mice (14) prompted us to explore the virulence potential of *G. parasuis* strain 174 (SV7) in mice. Bacteria that have host-specific transferrin receptors are often limited in their ability to replicate and cause infection in mice. This is illustrated by the requirement for human and not bovine transferrin or lactoferrin as an exogenous iron source for establishing a mouse model for sepsis and invasion infection by the human pathogen *Neisseria meningitidis* (15). For our experiment we opted to use iron dextran as an exogenous source of iron for growth of *G. parasuis* in lieu of porcine transferrin.

**Figure 3:**
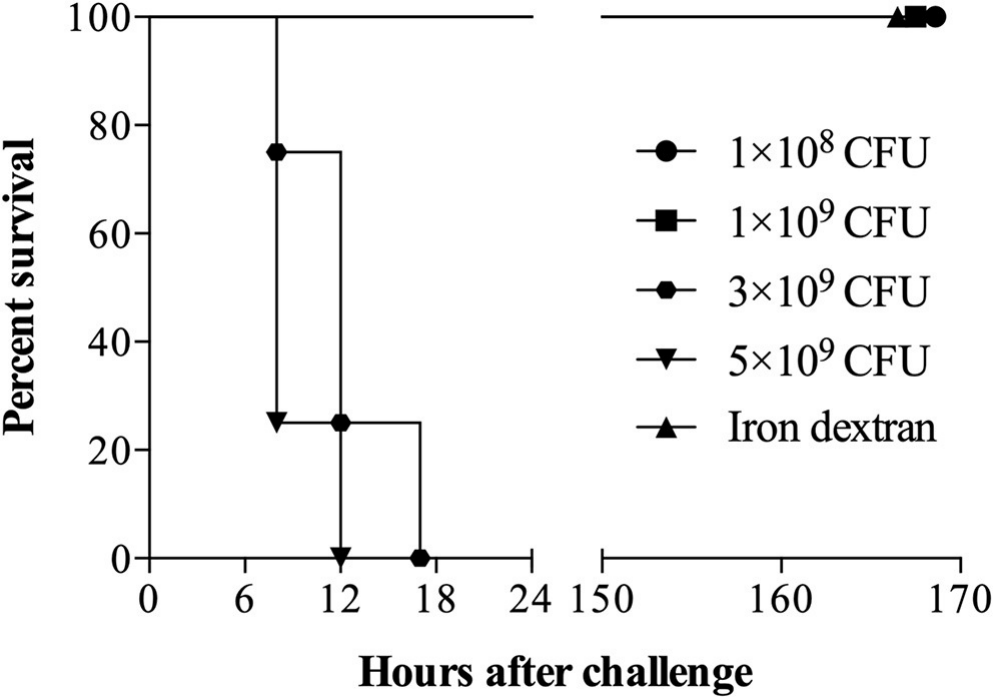
Survival of mice challenged intraperitoneally with *G. parasuis* strain 174 (serovar 7). Sixteen C57BL/6 mice (*n* = 4 per group) were challenged with 1 × 10^8^, 1 × 10^9^, 3 × 10^9^, and 5 × 10^9^ colony unit forming (CFU) of *G. parasuis* strain 174. The fifth group received only an intraperitoneal injection of iron dextran and served as sentinel control.

Groups of 5 mice were challenged by intraperitoneal injection of relatively high bacterial numbers (1 × 10^8^, 1 × 10^9^, 3 × 10^9^ or 5 × 10^9^) of *G. parasuis* strain 174 (SV7) and 3.2 mg of iron dextran. Challenge doses of up to 1 × 10^9^ bacteria did not affect the survival of the mice but death occurred within 24 h for mice challenged with the higher doses (Figure 3). The observation that none of the mice challenged with 1 × 10^9^ bacteria died whereas all the mice with 3 and 5 times the bacterial challenge dose rapidly developed signs and were euthanized, suggests that their deaths may be attributed to the response to lipooligosaccharide (LOS).

## Discussion

The recent occurrence of outbreaks of Glässer's disease with serovar 7 strains of *G. parasuis* in Chinese and Brazilian swine herds (4, 5) raise the question whether there has been an increase in virulence in strains of this serovar. This could potentially be explained by acquisition of virulence factors from more virulent strains by horizontal genetic exchange, which would readily be achieved by the efficient natural transformation system present in these species (16, 17). Although it is possible that the outbreaks in China and Brazil are related due to exchanges of animals between the two countries, the positive nasal cultures of *G. parasuis* reported earlier in GD cases in Australia (6) would tend to suggest that acquisition of virulence genes by strains of this serovar may be occurring more frequently. In the line of horizontal gene transfers, a novel *Glaesserella* sp. strain was recently described in Australia; this strain with high capacity to produce lung disease, has a mosaic genomic structure composed by virulence factors commonly identified in other members of the Pasteurellacea family, as *A. pleuropneumoniae, A. minor* and *P. multocida* (18). This results just highlight the genomic adaptive capacity of the disease causative microorganisms and in order to explore an alternate explanation for the GD cases caused by SV7 we decided to re-evaluate whether the reference strain for SV7 is really avirulent.

In this study we demonstrated that *G. parasuis* reference SV7 strain 174 is able to cause disease manifestations typical for GD thus should not be considered avirulent. This contrasts the results in the foundational study evaluating the virulence potential of the different serovars of *G. parasuis* (2) but could readily be explained by loss of virulence during passage. The pathogenesis of infection by the human pathogens *N. meningitidis* and *H. influenzae* has been studied extensively for over 30 years and has revealed genetic mechanisms for variation such as slip-strand mispairing (19) that has been shown to influence many genes that are important for the pathogenesis of infection (20–22). Phase variation by this process commonly occurs in frequencies of 10^−3^ or 10^−4^ (19). For a number of the phase variable factors that are modulated through slip-strand mispairing there is a tendency to select for non-expression during propagation in the laboratory, which can result in a reduction in virulence of the bacterial population. Phase variation has also been described in pathogens of food production animals such as *H. somni* (23) and likely is present in many pathogens from the Pasteurellaceae family, including *G. parasuis*.

The potential for loss of virulence during passage in the laboratory was the rationale for passaging the original avirulent SV7 strain through pigs in order to generate a more virulent version of the strain. In addition, the generation of a standard inoculum rather than selecting colonies for future experiments is based on the premise that a stock of a strain that has been passaged in the laboratory may be heterogeneous with respect to its virulence such that selection of individual colonies for challenge experiments may provide variable results. There can be very strong selective pressures for expression during passage in the host as demonstrated with the *opa* genes in the human pathogen *N. meningitidis* when used to colonize transgenic mice expressing the human CEACAM receptor that is required for efficient colonization (24). The effective selection for expression of phase variable loci required for survival and disease progression, or selection of variants that are generated by other mechanisms, is one of the more likely explanations for the observed increase in virulence of this the SV7 strain reported in this study.

Although an alternate explanation could be that the strain picked up virulence determinants from bacteria already present in the host, the disease progression in the pigs that were used to passage the strain was relatively rapid, making it unlikely that this had occurred in all the pigs in which the inoculum was administered into the lung through the trachea. The likelihood of acquiring virulence determinants from other bacteria during passage could have been further reduced had we administered the bacteria directly into the peritoneal cavity or the blood but also may not have provided the same opportunity for selection of phase variants and acquiring a more virulent strain. The potential to generate more virulent stocks of strains by passage through pigs might apply to some other non-virulent strains, which may be of value when the cross-protective properties of vaccines targeting these bacteria is being evaluated.

Finally, the clinical findings in this study are similar to those reported for GD caused by other serovars in that the clinical course was constant fever, lameness, respiratory distress, and in some cases, neurological signs (7, 8, 25–27). However, this study is the first to report on GD by in pigs infected with *G. parasuis* presenting with signs of eye congestion and corneal opacity. Lastly, the lymphoid depletion observed in the thymus can potentially explain the depletion of the TCRγδ^+^ lymphocytes during the systemic infection of *G. parasuis*, as previously reported by our group (28).

## Conclusion

We have demonstrated that *G. parasuis* SV7 strain 174, previously considered an avirulent strain, is capable of causing Glässer's disease (GD) in pigs. This strain causes severe clinicals signs that develop rapidly after challenge and can result in novel macroscopic lesions associated with GD. Our findings indicate that *G. parasuis* strain 174 should be considered a virulent serovar and that some caution should be used in employing vaccine strategies based on association between capsular serovar and virulence.

## Data Availability Statement

The datasets generated for this study are available on request to the corresponding author.

## Ethics Statement

This animal study was reviewed and approved by Institutional Committee for Ethical Use of Animals of the University of Passo Fundo and the Animal Care Committee of the University of Calgary.

## Author Contributions

RF, AS, and CD conceived and designed the experiments and analysis. CD, JG, SP, LK, SC, and RF performed *in vivo* experiments and microbiologic analysis. CD and DD conducted the pathological analysis. AS, RF, and CD analyzed the full data and wrote the paper.

### Conflict of Interest

The authors declare that the research was conducted in the absence of any commercial or financial relationships that could be construed as a potential conflict of interest.
